# Factors influencing abnormal cleavage of early embryos: time-lapse imaging analysis of 138,178 normally fertilized embryos

**DOI:** 10.3389/fendo.2026.1754938

**Published:** 2026-05-15

**Authors:** Weiwei Liu, Jun Shuai, Yang Gao, Qi Zhang, Xiaoni Guo, Wei Han, Guoning Huang

**Affiliations:** Women and Children’s Hospital of Chongqing Medical University, Chongqing Health Center for Women and Children, Chongqing, China

**Keywords:** abnormal cleavage, assisted reproductive technology, cleavage patterns, embryo, time-lapse Imaging

## Abstract

**Backgrounds:**

Abnormal cleavage (ABNCL) in early human embryos is a frequent phenomenon associated with impaired developmental competence and reduced reproductive potential. However, the clinical determinants of ABNCL and its subtypes remain insufficiently defined. This study aimed to identify patient- and treatment-related factors associated with ABNCL in embryos generated through *in vitro* fertilization.

**Methods:**

This retrospective cohort included 138,178 normally fertilized embryos cultured in a time-lapse imaging (TLI) system, comprising 77,260 normal cleavage (NC) and 60,918 ABNCL embryos. Univariate, stratified, and multivariable logistic regression with generalized estimating equation (GEE) analyses were performed to evaluate the associations between clinical characteristics—including demographics, controlled ovarian stimulation (COS) parameters, and insemination methods—and ABNCL occurrence and its subtypes. ABNCL was classified into direct cleavage (DC; including DC1 and DC2), rapid cleavage (RC), chaotic cleavage (CC), and other atypical patterns according to established morphokinetic criteria.

**Results:**

Among the 138,178 embryos analyzed, 55.9% exhibited NC and 44.1% demonstrated ABNCL. Stratified analyses revealed significant variability in ABNCL rates across baseline characteristics and stimulation-related factors. In GEE models, endometriosis, unexplained infertility, higher oocyte yield, intracytoplasmic sperm injection (ICSI), and rescue ICSI were independently associated with an increased risk of ABNCL. Conversely, the use of a mild stimulation protocol and higher estradiol (E2) levels on the hCG trigger day were linked to a reduced risk. Subtype analyses showed distinct factor-specific patterns: higher total gonadotropin (Gn) doses increased the likelihood of DC and “other” ABNCL types, while elevated initial Gn doses were associated with higher risks of RC and CC. Additionally, the gonadotropin-releasing hormone antagonist protocol specifically increased the risks of RC and “other” ABNCL patterns.

**Conclusions:**

ABNCL is a common event during early embryo development and is significantly influenced by both patient characteristics and treatment strategies. Endometriosis, unexplained infertility, higher oocyte yield, ICSI, and rescue ICSI were associated with increased ABNCL risk, whereas mild stimulation and higher E2 levels on the trigger day appeared protective. These findings underscore the importance of individualized COS and fertilization strategies to promote optimal early embryo development in assisted reproductive technology; however, their implications are limited to cleavage-stage outcomes and should not be extrapolated to later developmental or clinical endpoints.

## Introduction

Over the four decades since the first IVF birth, assisted reproductive technology (ART) has achieved major advances in controlled ovarian stimulation (COS), oocyte retrieval, embryo culture systems, and embryo selection strategies (1). Nevertheless, pregnancy and live birth rates worldwide remain suboptimal, and impaired early embryo developmental competence remains a major contributor to ART failure (2). Increasing evidence indicates that abnormal cleavage (ABNCL)—a qualitative disruption of early mitotic events—is strongly associated with compromised embryo development, reduced blastocyst formation, and lower implantation and pregnancy rates (3–6).

Conventional static morphological assessment often fails to capture the dynamic nature of early embryonic divisions, resulting in underdiagnosis of cleavage abnormalities (7). With the widespread adoption of time-lapse imaging (TLI), continuous morphokinetic monitoring now allows more accurate identification of aberrant cleavage phenomena such as direct cleavage (DC), rapid cleavage (RC), and chaotic cleavage (CC) (8–10). These abnormal patterns have consistently been linked to impaired blastocyst development, chromosomal instability, and poorer clinical outcomes (3, 11).

Despite these advances, current research has primarily focused on the downstream developmental consequences of ABNCL (12–16), whereas the upstream determinants—particularly clinical, endocrine, and treatment-related factors—remain insufficiently characterized. Existing studies are limited by small sample sizes, evaluation of only single ABNCL subtypes, or incomplete consideration of key variables such as infertility etiology, COS protocol, gonadotropin dosage, hormonal milieu, and insemination method. For example, Ferraretto et al. analyzed 1,343 embryos and found that embryo- or cycle-level factors explained 12–25% of the variance in early cleavage anomalies, but COS and endocrine parameters were not assessed (17). Burruel et al. demonstrated associations between sperm oxidative stress and early cleavage errors but did not account for maternal characteristics or stimulation-related factors (4). Moreover, systematic reviews highlight that the potential influence of COS intensity, gonadotropin exposure, peak estradiol (E2) levels, and oocyte yield on ABNCL remains largely unexplored (18, 19). Even large contemporary TLI studies, such as Shavit et al., have focused predominantly on developmental potential rather than identifying determinants of ABNCL (6).

Given these gaps, a comprehensive evaluation of patient characteristics, stimulation strategies, endocrine profiles, and insemination methods in relation to ABNCL is critically needed. Such evidence is essential for clarifying the biological and iatrogenic contributors to abnormal early embryogenesis and has direct implications for individualized ART treatment planning. Therefore, leveraging a large real-world cohort of 138,178 normally fertilized embryos monitored via TLI, this study systematically investigates the clinical and treatment-related determinants of ABNCL and its subtypes. This work aims to fill a major knowledge gap in early embryo biology and support evidence-based optimization of COS strategies and embryo management in ART.

## Materials and methods

### Study population

**Figure 1:**
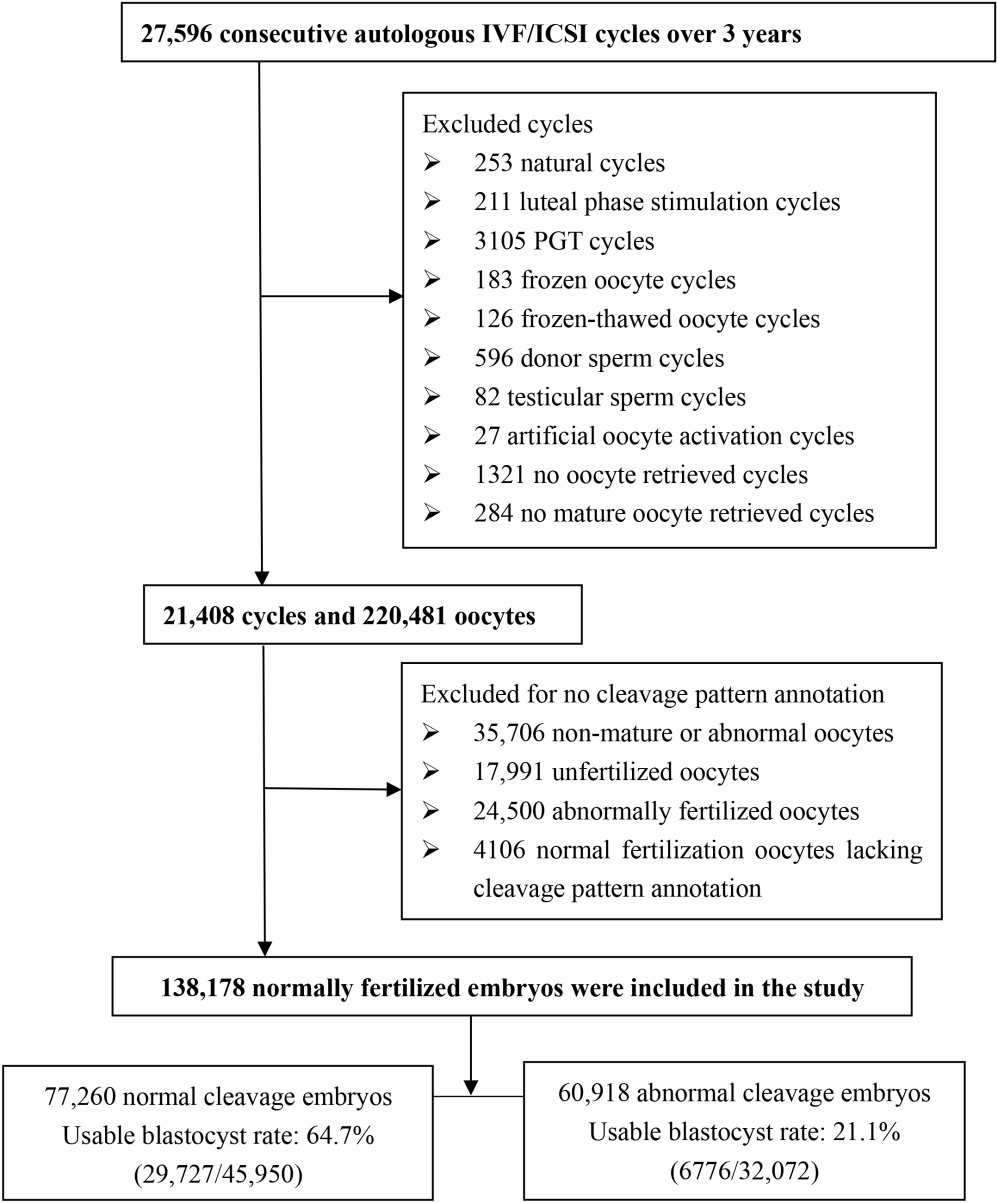
Flowchart of embryos included in the study.

This retrospective cohort study included 21,408 consecutive autologous IVF and intracytoplasmic sperm injection (ICSI) cycles performed at the Center for Reproductive Medicine, Women and Children’s Hospital of Chongqing Medical University (Chongqing, China) between January 2021 and December 2023. We excluded natural cycles, luteal phase stimulation cycles, preimplantation genetic testing (PGT) cycles, frozen oocyte cycles, frozen-thawed oocyte cycles, donor sperm cycles, testicular sperm cycles, artificial oocyte activation cycles, no oocyte retrieved cycles, and no mature oocyte retrieved cycles. In addition, immature, abnormal, unfertilized, and abnormally fertilized oocytes were excluded because they lacked cleavage pattern annotation. Furthermore, normal fertilized oocytes lacking cleavage pattern annotation due to imaging obstructions caused by improper petri dish placement or small bubbles in the culture medium were excluded. **Figure 1** displays a flowchart of the analyzed embryos. Retrospective data analysis was approved by the Clinical Application and Ethics Committee of Human Assisted Reproductive Technology in Chongqing Health Center for Women and Children (2024-RGI-18), and the requirement for informed consent was waived in light of its retrospective design.

### Clinical factors assessed for impact on abnormal cleavage

Data and information regarding patients were retrieved from the reproductive medicine center’s database. The clinical factors analyzed in this study included: female age, female body mass index (BMI), infertility type (primary infertility and secondary infertility), infertility factors (tubal factors, decreased ovarian reserve [DOR], endometriosis [EMT], ovulatory disorders, male factors, and other unexplained infertility factors), sperm morphology (normal and abnormal, classified according to the WHO [5th edition] criteria), COS protocol (gonadotropin-releasing hormone [GnRH] agonist protocol, GnRH antagonist protocol, and mild stimulation protocol), gonadotropin (Gn) type (recombinant Gn and urinary-derived Gn), Gn initial dose, ovarian stimulation duration, total Gn dose, E2 level on hCG trigger day, number of oocytes retrieved, and insemination method (IVF, ICSI, and rescue ICSI).

### Gamete preparation

All patients received tailored COS protocols designed by experienced physicians, according to their individual baseline characteristics and ART history. We previously described the GnRH agonist protocol and GnRH antagonist protocol (20). For patients undergoing mild stimulation, the ovarian stimulation protocol included daily administration of 50 mg of clomiphene citrate (Merck) and 150 IU recombinant human follicle-stimulating hormone (rFSH, Livzon, China) or urinary follicle-stimulating hormone (uFSH, Jinsai, China) initiated on day 2 or day 3 of the menstrual cycle. When the leading follicle was ≥16 mm, 0.2 mg of triptorelin acetate (Decapeptyl, Ferring, Switzerland) and 2000 IUs of hCG (Ovidrel Merck, Germany) were injected to trigger oocyte maturation and ovulation.

Transvaginal oocyte aspiration and sperm preparation were performed via IVF, ICSI, or early rescue ICSI, as described previously (21, 22). Oocyte-cumulus complexes (OCCs) were washed and maintained in G-IVF Plus media (Vitrolife, Sweden) at 6% CO_2_ and 37 °C until completion of oocyte collection. The OCCs were then transferred to culture dishes containing G-IVF Plus media (Vitrolife) overlaid with Ovoil (Vitrolife) and incubated at 37 °C with 6% CO_2_ and 5% O_2_ before conventional IVF insemination or denudation for ICSI.

### Fertilization and embryo culture

For patients undergoing IVF fertilization, prepared sperm were co-incubated with OCCs for 4 to 6 hours until the second polar body was extruded from the oocyte, indicating oocyte activation, which was assessed after cumulus cell removal. Then, the activated oocytes were transferred to culture dishes containing G1 Plus media (Vitrolife) overlaid with Ovoil (Vitrolife) and incubated at 37 °C with 6% CO_2_ and 5% O_2_. Non-activated oocytes were incubated overnight in G-IVF Plus media (Vitrolife) with prepared sperm, after which oocytes confirmed as having two pronuclei (2PN) were transferred to G1 Plus media (Vitrolife) and placed into the EmbryoScope+ for culturing until Day 3.

Oocytes intended for insemination by ICSI were denuded by exposure to 80 IU/ml Hyadase (Dewin, China) for a maximum of 1 min, followed by mechanical removal of cumulus cells in G-MOPS (Vitrolife). Oocytes at the metaphase II stage were injected with a single spermatozoon and subsequently transferred to G1 Plus media (Vitrolife) and loaded into the EmbryoScope+ incubator (Vitrolife) for culturing until Day 3 at 37 °C with 6% CO_2_ and 5% O_2_.

If oocytes intended for patients undergoing IVF fertilization did not show a second polar body within 4 to 6 hours of cumulus cells removal, or if the proportion of second polar bodies was less than one-third of the mature oocytes, then the mature oocytes lacking a second polar body were subjected to early rescue ICSI and subsequently transferred to G1 Plus media (Vitrolife) and loaded into the EmbryoScope+ incubator (Vitrolife) for culturing until Day 3 at 37 °C with 6% CO_2_ and 5% O_2_.

Embryos were scored at Day 3 according to ESHRE consensus criteria (23). Meanwhile, the day 3 morphological assessment, together with the cleavage pattern parameters observed in TLI, offered a comprehensive evaluation of the embryos. All evaluations were conducted by experienced embryologists following standardized protocols, with routine quality control measures implemented to ensure inter-observer consistency. Subsequent clinical decisions, including embryo transfer, vitrification, or extended culture, were based on embryo quality and patient-specific factors. If blastocyst culture was needed, embryos were cultured in G2 Plus media and incubated in the EmbryoScope+ incubator (Vitrolife) from Day 3 to Day 7.

### Definition of abnormal cleavage

Based on the developmental status of embryos as determined by TLI, fertilized embryos were classified as normal cleavage (NC) or ABNCL embryos. NC and ABNCL events were annotated from the first mitotic division up to Day 2. NC refers to normally fertilized eggs that divided from one parent cell into two daughter cells during the first mitotic division, followed by division of either of the two cells continues into two daughter cells during the second mitotic division. ABNCL was further classified into the following five types: Direct cleavage type 1(DC1): abnormal first mitotic division, where normally fertilized eggs divided directly into three or more daughter cells; Direct cleavage type 2 (DC2): abnormal second mitotic division, where, after the first division, the normally fertilized egg divided into two cells, and one or both of these cells then divided into three or more daughter cells during the second round of division; Rapid cleavage (RC): The normally fertilized egg divided into two cells during the first mitotic division, then quickly divided into three cells, with the two-cell stage lasting no longer than 5 hours; Chaotic cleavage (CC): the normally fertilized egg displayed uneven cleavage and fragmentation; Other: the normally fertilized eggs did not divide, divided after 48 hours of culturing, or underwent reverse cleavage. Embryo cleavage pattern annotation was completed by three embryologists with over five years of work experience after rigorous training. The inter-observer agreement for cleavage pattern assessment was evaluated in a subset of 200 normally fertilized embryos using Fleiss’ kappa (κ). The analysis demonstrated perfect agreement (κ=1) for the NC/ABNCL dichotomy and almost perfect agreement (κ=0.975) for the subtype of ABNCL.

### Statistical analysis

The statistical package SPSS 21.0 (IBM Corp., USA) was used to perform all statistical analyses. Continuous variables are expressed as mean and SD, whereas categorical variables are described using percentages, and one-way analysis was performed using the Chi-squared test for comparisons. Independent samples t-test was used for comparisons between two groups. Standardized mean difference (SMD) was used to evaluate the magnitude of baseline differences in light of the large cohort size. A multivariable logistic regression with generalized estimating equation (GEE) model was employed to assess the association between clinical factors and ABNCL, accounting for the clustering of multiple normally fertilized oocytes and embryos within each patient. Clinical variables that demonstrated statistical significance in univariate analyses were included in the GEE model. These variables included female age, BMI, infertility type, infertility factor, sperm morphology, COS protocol, Gn initial dose, Gn duration, total Gn dose, E2 level on hCG trigger day, number of oocytes retrieved, and insemination method. Odds ratios (ORs) with corresponding 95% confidence intervals (CIs) and P-values were reported for each variable.

The statistical power was calculated based on the sample size and effect estimates, yielding a value of 1.0. To assess potential multicollinearity among continuous stimulation-related variables—Gn initial dose, Gn duration, total Gn dose, E2 level on hCG trigger day, and number of oocytes retrieved—variance inflation factor (VIF) diagnostics were conducted. All VIF values were below 5, indicating no evidence of significant multicollinearity among these predictors.

## Results

### Participants’ baseline characteristics and clinical factors associated with abnormal cleavage

The baseline characteristics of the study participants are shown in **Table 1**. In total, 138,178 normally fertilized embryos were included in the study. Due to the large sample size, many variables included female age, BMI, infertility type, infertility factor, sperm morphology, COS protocol, Gn initial dose, Gn duration, total Gn dose, E2 level on hCG trigger day, insemination method, number of oocytes retrieved and Day 3 usable embryo rate had statistically significant (*p*<0.001). However, the SMD was below 0.1 for most parameters, indicating good overall balance. A moderate imbalance was noted in the insemination method (SMD = 0.233). An expected difference was observed in the Day 3 usable embryo rate (SMD = 0.596), which was intrinsically lower in the ABNCL group.

**Table 1:** Baseline characteristics of the two groups.

Variables	Total	ABNCL	NC	*P*	SMD
Embryos (n)	138,178	60,918	77,260	–	
Female age (years)	31.52 ± 4.35	31.46 ± 4.29	31.57 ± 4.40	<0.001**	0.025
BMI (kg/m^2^)	22.70 ± 3.27	22.68 ± 3.26	22.72 ± 3.28	0.013*	0.012
Infertility type				<0.001**	0.046
Primary infertility	48.8% (65,476/13,4203)	50.1% (29,328/58,564)	47.8% (36,148/75,639)		
Secondary infertility	51.2% (68,727/13,4203)	49.9% (29,236/58,564)	52.2% (39,491/75,639)		
AMH (ng/ml)	4.38 ± 2.99	4.36 ± 2.90	4.39 ± 3.06	0.095	0.010
AFC	9.94 ± 5.30	9.95 ± 5.22	9.94 ± 5.36	0.672	0.002
Indication for infertility					
Tubal factor	77.8% (107,489/138,178)	75.6% (46,028/60,918)	79.6% (61,461/77,260)	<0.001**	0.096
DOR	6.4% (8840/138,178)	6.1% (3739/60,918)	6.6% (5101/77,260)	<0.001**	0.020
EMT	14.3% (19,699/138,178)	14.5% (8838/60,918)	14.1% (10,861/77,260)	0.017*	0.011
Ovulatory disorder	12.8% (17,731/138,178)	12.0% (7299/60,918)	13.5% (10,432/77,260)	<0.001**	0.044
Male factor	16.2% (22,387/138,178)	17.9% (10,933/60,918)	14.8% (11,454/77,260)	<0.001**	0.084
Unexplained infertility	7.8% (10,812/138,178)	8.8% (5375/60,918)	7.0% (5437/77,260)	<0.001**	0.067
Semen morphology				<0.001**	0.049
Normal	91.4% (122,340/133,858)	90.6% (53,210/58,714)	92.0% (69,130/75,144)		
Teratozoospermia	8.6% (11,518/133,858)	9.4% (5504/58,714)	8.0% (6014/75,144)		
COS regimen				<0.001**	0.058
GnRH agonist	47.4% (65,473/138,178)	46.9% (28,578/60,918)	47.8% (36,895/77,260)		
GnRH antagonist	48.8% (67,431/138,178)	49.8% (30,323/60,918)	48.0% (37,108/77,260)		
Mild stimulation	3.8% (5274/138,178)	3.3% (2017/60,918)	4.2% (3257/77,260)		
Type of Gn				0.080	0.013
r-FSH	84.1% (115,917/137,814)	83.9% (51,015/60,792)	84.3% (64,902/77,022)		
u-FSH	15.9% (21,897/137,814)	16.1% (9777/60,792)	15.7% (12,120/77,022)		
Initial Gn dosage (IU)	193.06 ± 55.25	193.59 ± 55.03	192.64 ± 55.42	0.002**	0.017
Duration of ovarian stimulation (days)	9.15 ± 1.79	9.16 ± 1.75	9.14 ± 1.82	0.011*	0.011
Total Gn dosage (IU)	1832.55 ± 668.03	1842.16 ± 664.56	1824.96 ± 670.67	<0.001**	0.026
E2 level on hCG trigger day (pg/mL)	2779.24 ± 1361.82	2769.26 ± 1346.04	2787.11 ± 1374.10	0.015*	0.013
Progesterone level on hCG trigger day (ng/mL)	0.67 ± 0.56	0.67 ± 0.55	0.67 ± 0.56	0.090	0.000
Oocyte retrieval	15.23 ± 7.76	15.34 ± 7.63	15.14 ± 7.86	<0.001**	0.026
Insemination method				<0.001**	0.233
IVF	72.3% (99,871/138,178)	66.9% (40,735/60,918)	76.5% (59,136/77,260)		
ICSI	24.8% (34,238/138,178)	29.8% (18,152/60,918)	20.8% (16,086/77,260)		
Rescue ICSI	2.9% (4069/138,178)	3.3% (2031/60,918)	2.6% (2038/77,260)		
Usable embryo rate on Day 3 (%)	54.46 ± 23.45	47.04 ± 22.61	60.31 ± 22.43	<0.001**	0.596

ABNCL, abnormal cleavage; NC, normal cleavage; BMI, body mass index; AMH, anti-Müllerian hormone; AFC, antral follicle count; DOR, diminished ovarian reserve; EMT, endometriosis; COS, controlled ovarian stimulation; GnRH, gonadotropin-releasing hormone; Gn, gonadotropin; r-FSH, recombinant follicle stimulating hormone; u-FSH, urinary follicle stimulating hormone; E2, estradiol; hCG, human chorionic gonadotropin; IVF, in vitro fertilization; ICSI, intracytoplasmic sperm injection; SMD, standardized mean difference.

### Subtype distribution and stratified univariate analysis of abnormal cleavage

To partially control for the confounding effects of clinical variables, we performed stratified univariate analyses of the incidence rates of different ABNCL subtypes based on baseline characteristics with statistical significance in **Table 1**. Overall, NC and ABNCL embryos accounted for 55.9% (77,260/138,178) and 44.1% (60,918/138,178), respectively. The incidence rates of the five ABNCL subtypes were as follows: DC1 (9.0%), DC2 (9.0%), RC (10.2%), CC (4.5%), and other (11.4%). Stratified univariate analyses showed that among all evaluated factors, only the type of Gn administered (r-FSH vs. u-FSH) was not significantly associated with ABNCL rates across subgroups. In contrast, significant differences in ABNCL rates were observed across subgroups defined by female age, BMI, infertility type, indication for infertility, semen morphology, COS protocol, initial Gn dose, Gn duration, total Gn dose, E2 level on hCG trigger day, number of oocytes retrieved, and insemination method (all *p* < 0.001). The detailed associations between these clinical factors and ABNCL rates are presented in **Table 2**.

**Table 2:** Subtype distribution and stratified univariate analysis of ABNCL.

Variables	ABNCL					NC No. (%)	*P*
	DC1 No. (%)	DC2 No. (%)	RC No. (%)	CC No. (%)	Other No. (%)		
Embryos (n=138, 178)	12456 (9.0)	12390 (9.0)	14,162 (10.2)	6226 (4.5)	15,684 (11.4)	77,260 (55.9)	
Female age (years)							<0.001**
<35 (n=105, 520)	9655 (9.1)	9551 (9.1)	10,846 (10.3)	4807 (4.6)	11,944 (11.3)	58,717 (55.6)	
35–39 (n=26, 297)	2315 (8.8)	2326 (8.8)	2675 (10.2)	1179 (4.5)	3051 (11.6)	14,751 (56.1)	
≥40 (n=6361)	486 (7.6)	513 (8.1)	641 (10.1)	240 (3.8)	689 (10.8)	3792 (59.6)	
BMI (kg/m^2^)							<0.001**
≤18.5 (n=9713)	983 (10.1)	871 (9.0)	962 (9.9)	434 (4.5)	1083 (11.2)	5380 (55.4)	
18.6–24.9 (n=97, 402)	8963 (9.2)	8735 (9.0)	10083 (10.4)	4400 (4.5)	10,860 (11.1)	54,361 (55.8)	
≥25 (n=31,009)	2506 (8.1)	2781 (9.0)	3117 (10.1)	1392 (4.5)	3738 (12.1)	17,475 (56.4)	
Infertility type							<0.001**
Primary infertility (n=65, 476)	6001 (9.2)	5833 (8.9)	6930 (10.6)	2986 (4.6)	7578 (11.6)	36,148 (55.2)	
Secondary infertility (n=68, 727)	6016 (8.8)	6121 (8.9)	6660 (9.7)	2990 (4.4)	7449 (10.8)	39,491 (57.5)	
Indication for infertility							
Tubal factor (n=107,489)	9679 (9.0)	9479 (8.8)	10,732 (10.0)	4594 (4.3)	11,544 (10.7)	61,461 (57.2)	<0.001**
DOR (n=8840)	807 (9.1)	717 (8.1)	899 (10.2)	352 (4.0)	964 (10.9)	5101 (57.7)	<0.001**
EMT (n=19,699)	1720 (8.7)	1756 (8.9)	2077 (10.5)	959 (4.9)	2326 (11.8)	10,861 (55.1)	<0.001**
Ovulatory disorder (n=17,731)	1491 (8.4)	1465 (8.3)	1727 (9.7)	658 (3.7)	1958 (11.0)	10432 (58.8)	<0.001**
Male factor (n=22, 387)	2068 (9.2)	2070 (9.2)	2610 (11.7)	1167 (5.2)	3018 (13.5)	11,454 (51.2)	<0.001**
Unexplained infertility (n=10, 812)	969 (9.0)	1036 (9.6)	1231 (11.4)	613 (5.7)	1526 (14.1)	5437 (50.3)	<0.001**
Semen morphology							<0.001**
Normal (n=122, 340)	11,017 (9.0)	10,915 (8.9)	12,321 (10.1)	5418 (4.4)	13,539 (11.1)	69,130(56.5)	
Teratozoospermia (n=11, 518)	958 (8.3)	1064 (9.2)	1354 (11.8)	582 (5.1)	1546 (13.4)	6014 (52.2)	
COS regimen							<0.001**
GnRH agonist (n=65, 473)	6076 (9.3)	6071 (9.3)	6275 (9.6)	3089 (4.7)	7067 (10.8)	36,895 (56.4)	
GnRH antagonist (n=67, 431)	6003 (8.9)	5919 (8.8)	7323 (10.9)	2979 (4.4)	8099 (12.0)	37108 (55.0)	
Mild stimulation (n=5274)	377 (7.1)	400 (7.6)	564 (10.7)	158 (3.0)	518 (9.8)	3257 (61.8)	
Type of Gn							0.167
r-FSH (n=115, 917)	10479 (9.0)	10409 (9.0)	11784 (10.2)	5215 (4.5)	13128 (11.3)	64902 (56.0)	
u-FSH (n=21, 897)	1957 (8.9)	1958 (8.9)	2349 (10.7)	998 (4.6)	2515 (11.5)	12120 (55.4)	
Initial Gn dosage (IU)							<0.001**
≤150 (n=65, 175)	5843 (9.0)	5920 (9.1)	6686 (10.3)	2706 (4.2)	7113 (10.9)	36,907 (56.6)	
151–299 (n=56, 126)	5068 (9.0)	5041 (9.0)	5893 (10.5)	2722 (4.8)	6546 (11.7)	30856 (55.0)	
≥300 (n=16, 877)	1545 (9.2)	1429 (8.5)	1583 (9.4)	798 (4.7)	2025 (12.0)	9497 (56.3)	
Duration of ovarian stimulation (days)							<0.001**
<10 (n=81, 516)	7324 (9.0)	7191 (8.8)	8705 (10.7)	3533 (4.3)	9397 (11.5)	45366 (55.7)	
10–11 (n=46, 558)	4174 (9.0)	4246 (9.1)	4525 (9.7)	2214 (4.8)	5082 (10.9)	26317 (56.5)	
>11 (n=10, 104)	958 (9.5)	953 (9.4)	932 (9.2)	479 (4.7)	1205 (11.9)	5577 (55.2)	
Total gonadotropin dosage (IU)							<0.001**
≤1500 (n=53, 547)	4683 (8.7)	4816 (9.0)	5777 (10.8)	2214 (4.1)	5800 (10.8)	30,257 (56.5)	
1501–2000 (n=32, 014)	2889 (9.0)	2864 (8.9)	3390 (10.6)	1487 (4.6)	3582 (11.2)	17802 (55.6)	
>2000 (n=52, 617)	4884 (9.3)	4710 (9.0)	4995 (9.5)	2525 (4.8)	6302 (12.0)	29,201 (55.5)	
E2 level on hCG trigger day (pg/mL)							<0.001**
< 2000 (n=45, 953)	4090 (8.9)	4125 (9.0)	4817 (10.5)	1951 (4.2)	5237 (11.4)	25,733 (56.0)	
2000–3999 (n=60885)	5513 (9.1)	5517 (9.1)	6250 (10.3)	2907 (4.8)	7056 (11.6)	33,642 (55.3)	
≥4000 (n=31, 193)	2837 (9.1)	2729 (8.7)	3086 (9.9)	1361 (4.4)	3378 (10.8)	17,802 (57.1)	
Oocyte retrieval							<0.001**
<6 (n=12, 611)	974 (7.7)	1047 (8.3)	1271 (10.1)	434 (3.4)	1260 (10.0)	7625 (60.5)	
6–15 (n=65, 374)	5917 (9.1)	5952 (9.1)	6594 (10.1)	3072 (4.7)	7619 (11.7)	36,220 (55.4)	
>15 (n=60, 193)	5565 (9.2)	5391 (9.0)	6297 (10.5)	2720 (4.5)	6805 (11.3)	33,415 (55.5)	
Insemination method							<0.001**
IVF (n=99, 871)	8712 (8.7)	8866 (8.9)	9295 (9.3)	3908 (3.9)	9954 (10.0)	59,136 (59.2)	
ICSI (n=34, 238)	3364 (9.8)	3192 (9.3)	4353 (12.7)	2081 (6.1)	5162 (15.1)	16086 (47.0)	
Rescue ICSI (n=4069)	380 (9.3)	332 (8.2)	514 (12.6)	237 (5.8)	568 (14.0)	2038 (50.1)	

ABNCL, abnormal cleavage; NC, normal cleavage; DC1, direct cleavage type 1; DC2, direct cleavage type 2; RC, rapid cleavage; CC, chaotic cleavage; BMI, body mass index; DOR, diminished ovarian reserve; EMT, endometriosis; COS, controlled ovarian stimulation; GnRH, gonadotropin-releasing hormone; Gn, gonadotropin; r-FSH, recombinant follicle stimulating hormone; u-FSH, urinary follicle stimulating hormone; E2, estradiol; hCG, human chorionic gonadotropin; IVF, in vitro fertilization; ICSI, intracytoplasmic sperm injection. ** p < 0.01.

### GEE analysis of factors associated with abnormal cleavage and its subtypes

To further examine the independent associations between clinical factors and ABNCL, a multivariable logistic GEE model was constructed, incorporating all variables identified as significant in univariate stratified analyses. Additionally, separate GEE models analyses were conducted for each of the five ABNCL subtypes.

Overall, EMT and unexplained infertility increased the risk of ABNCL, retrieving between six and fifteen oocytes, or more than fifteen oocytes, was associated with an increased risk of ABNCL compared to retrieving fewer than six oocytes. ICSI and rescue ICSI were linked to a higher risk of ABNCL than conventional IVF. Mild stimulation protected against ABNCL compared to GnRH agonist stimulation, while the GnRH antagonist protocol increased the risk of RC and other types of ABNCL compared to the GnRH agonist protocol. The hCG trigger day E2 level above 4000 pg/ml, compared to below 2000 pg/ml, protected against ABNCL. The initial Gn dose ranging from 150 to 300 IU, compared to doses lower than 150 IU, was a risk factor for both RC and CC. In addition, the total Gn dose exceeding 2000 IU, compared to the dose of 1500 IU or lower, was linked to an increased risk of DC1 and other types of ABNCL. Because Table 3 contains many types of data items, it cannot be fully displayed in one table, therefore, we divided it into **Table 3-1** and **Table 3-2**. The detailed GEE analysis of factors associated with ABNCL and its subtypes are presented in **Table 3-1** and **Table 3-2**.

**Table 3-1:** GEE analysis of factors associated with ABNCL and its subtypes.

Variables	Total ABNCL		DC1		DC2	
	Adjusted OR (95% CI)	*p*	Adjusted OR (95% CI)	*p*	Adjusted OR (95% CI)	*p*
Female age (35-39/<35)	0.971 (0.934-1.010)	0.142	0.920 (0.861-0.983)	0.013*	0.986 (0.927-1.049)	0.657
Female age (≥40/<35)	0.873 (0.811-0.939)	<0.001**	0.813 (0.713-0.928)	0.002**	0.906 (0.803-1.024)	0.113
BMI (18.6–24.9/≤18.5)	0.995 (0.940-1.054)	0.870	0.904(0.819-0.998)	0.045*	1.015 (0.926-1.112)	0.753
BMI (≥25/≤18.5)	0.986 (0.925-1.052)	0.676	0.778 (0.697-0.868)	<0.001**	1.017 (0.918-1.126)	0.747
Infertility type (Secondary infertility/Primary infertility)	0.950 (0.921-0.980)	0.001**	0.941 (0.894-0.990)	0.020*	0.990 (0.944-1.037)	0.658
Indication for infertility-Tubal	0.989 (0.935-1.046)	0.690	1.025 (0.933-1.125)	0.611	0.951 (0.872-1.037)	0.253
Indication for infertility-DOR	1.005 (0.942-1.072)	0.886	1.123(1.004-1.257)	0.043*	0.934 (0.841-1.038)	0.206
Indication for infertility-EMT	1.058 (1.017-1.101)	0.006**	0.956 (0.894-1.022)	0.186	1.025 (0.963-1.092)	0.436
Indication for infertility- Ovulatory disorder	0.978 (0.942-1.036)	0.656	0.957 (0.890-1.021)	0.177	0.982(0.897-1.062)	0.705
Indication for infertility-Male factor	0.966 (0.891-1.047)	0.400	1.026 (0.901-1.168)	0.697	0.975 (0.862-1.103)	0.691
Indication for infertility-Unexplained infertility	1.100 (1.016-1.191)	0.018*	0.939 (0.818-1.077)	0.367	1.028(0.911-1.161)	0.649
Semen morphology (Teratozoospermia/Normal)	1.020 (0.933-1.115)	0.663	0.833 (0.720-0.964)	0.014*	1.005 (0.880-1.148)	0.938
COS regimen (GnRH antagonist/agonist)	1.031 (0.992-1.071)	0.122	0.946(0.887-1.010)	0.094	0.985 (0.929-1.043)	0.601
COS regimen (Mild/agonist)	0.903 (0.819-0.996)	0.042*	0.799 (0.675-0.945)	0.009**	0.797 (0.681-0.932)	0.005**
Initial Gn dosage (151-299/≤150)	1.027 (0.981-1.075)	0.253	0.967 (0.896-1.044)	0.389	0.975(0.911-1.044)	0.469
Initial Gn dosage (≥300/≤150)	0.975 (0.907-1.046)	0.478	0.983 (0.872-1.108)	0.780	0.903 (0.808-1.008)	0.068
Duration of ovarian stimulation (10-11/<10)	0.965 (0.926-1.006)	0.093	0.884 (0.826-0.946)	<0.001**	1.002 (0.941-1.066)	0.961
Duration of ovarian stimulation (>11/<10)	1.032 (0.960-1.108)	0.393	0.947 (0.839-1.070)	0.381	1.073 (0.964-1.194)	0.198
Total gonadotropin dosage (1501-2000/≤1500)	0.983 (0.936-1.033)	0.501	1.084 (0.999-1.177)	0.053	0.964 (0.896-1.038)	0.336
Total gonadotropin dosage (>2000/≤1500)	1.030 (0.971-1.092)	0.326	1.178 (1.068-1.299)	0.001**	0.983 (0.898-1.076)	0.712
E2 level on hCG-trigger day (2000-3999/< 2000)	0.981 (0.945-1.019)	0.331	0.953 (0.894-1.017)	0.147	0.943 (0.889-1.000)	0.051
E2 level on hCG-trigger day (≥4000/< 2000)	0.908 (0.862-0.956)	<0.001**	0.924 (0.846-1.010)	0.082	0.870 (0.804-0.941)	<0.001**
Oocyte retrieval (6-15/<6)	1.208 (1.141-1.280)	<0.001**	1.261 (1.138-1.396)	<0.001**	1.121(1.019-1.234)	0.019*
Oocyte retrieval (>15/<6)	1.241 (1.161-1.326)	<0.001**	1.350 (1.200-1.518)	<0.001**	1.143 (1.025-1.275)	0.016*
Insemination method (ICSI/IVF)	1.594 (1.531-1.660)	<0.001**	1.404 (1.311-1.504)	<0.001**	1.284 (1.205-1.367)	<0.001**
Insemination method (rescue-ICSI/IVF)	1.426 (1.317-1.544)	<0.001**	1.261 (1.104-1.441)	<0.001**	1.074 (0.945-1.220)	0.274

* p < 0.05, ** p < 0.01.

**Table 3-2:** GEE analysis of factors associated with ABNCL and its subtypes.

Variables	RC		CC		Other	
	Adjusted OR (95% CI)	*p*	Adjusted OR (95% CI)	*p*	Adjusted OR (95% CI)	*p*
Female age (35-39/<35)	0.989 (0.929-1.052)	0.718	0.941 (0.852-1.039)	0.229	1.003 (0.942-1.067)	0.927
Female age (≥40/<35)	0.898 (0.799-1.009)	0.070	0.839 (0.696-1.010)	0.064	0.882 (0.781-0.996)	0.043*
BMI (18.6–24.9/≤18.5)	1.082 (0.989-1.184)	0.087	0.964 (0.836-1.112)	0.618	1.001 (0.911-1.100)	0.986
BMI (≥25/≤18.5)	1.063 (0.961-1.176)	0.233	0.997 (0.850-1.170)	0.972	1.075 (0.969-1.194)	0.172
Infertility type (Secondary infertility/Primary infertility)	0.913 (0.871-0.958)	<0.001**	0.955 (0.881-1.036)	0.267	0.953 (0.908-1.001)	0.055
Indication for infertility-Tubal	1.013 (0.930-1.105)	0.764	0.946 (0.821-1.090)	0.444	0.984 (0.899-1.077)	0.726
Indication for infertility-DOR	0.978 (0.884-1.083)	0.672	1.016 (0.861-1.198)	0.853	0.983 (0.883-1.095)	0.759
Indication for infertility-EMT	1.069 (1.004-1.139)	0.038*	1.117 (1.009-1.238)	0.034*	1.134 (1.063-1.209)	<0.001**
Indication for infertility- Ovulatory disorder	0.948 (0.881-1.020)	0.468	0.928 (0.820-1.051)	0.358	0.960 (0.880-1.048)	0.366
Indication for infertility-Male factor	0.972 (0.857-1.102)	0.657	0.853 (0.699-1.042)	0.120	0.937 (0.820-1.072)	0.344
Indication for infertility-Unexplained infertility	1.085 (0.957-1.230)	0.203	1.170 (0.962-1.425)	0.116	1.273 (1.126-1.440)	<0.001**
Semen morphology (Teratozoospermia/Normal)	1.053 (0.921-1.204)	0.447	1.098 (0.879-1.372)	0.409	1.105 (0.961-1.270)	0.162
COS regimen (GnRH antagonist/agonist)	1.131 (1.064-1.201)	<0.001**	0.953 (0.864-1.051)	0.334	1.105 (1.039-1.176)	0.002**
COS regimen (Mild/agonist)	1.069 (0.918-1.244)	0.391	0.760 (0.579-0.998)	0.049*	0.974 (0.813-1.168)	0.777
Initial Gn dosage (151-299/≤150)	1.122 (1.043-1.207)	0.002**	1.142 (1.013-1.287)	0.030*	1.003 (0.932-1.080)	0.933
Initial Gn dosage (≥300/≤150)	0.988 (0.879-1.110)	0.839	1.192 (0.993-1.430)	0.060	0.943 (0.840-1.059)	0.319
Duration of ovarian stimulation (10-11/<10)	0.999 (0.935-1.067)	0.972	1.035 (0.931-1.150)	0.528	0.943 (0.882-1.008)	0.082
Duration of ovarian stimulation (>11/<10)	1.058 (0.946-1.183)	0.324	1.056 (0.878-1.270)	0.565	1.031(0.920-1.155)	0.602
Total gonadotropin dosage (1501-2000/≤1500)	0.893 (0.825-0.965)	0.004**	0.966 (0.847-1.101)	0.606	1.017 (0.940-1.101)	0.676
Total gonadotropin dosage (>2000/≤1500)	0.849 (0.771-0.935)	<0.001**	0.988 (0.848-1.151)	0.874	1.153 (1.047-1.269)	0.004**
E2 level on hCG-trigger day (2000-3999/< 2000)	0.960 (0.904-1.020)	0.185	1.102 (0.999-1.215)	0.053	1.010 (0.951-1.073)	0.747
E2 level on hCG-trigger day (≥4000/< 2000)	0.867 (0.799-0.940)	<0.001**	1.032 (0.903-1.179)	0.646	0.925 (0.850-1.006)	0.069
Oocyte retrieval (6-15/<6)	1.143 (1.039-1.257)	0.006**	1.337 (1.141-1.565)	<0.001**	1.272 (1.154-1.402)	<0.001**
Oocyte retrieval (>15/<6)	1.227 (1.101-1.368)	<0.001**	1.277 (1.065-1.532)	0.008**	1.256 (1.123-1.404)	<0.001**
Insemination method (ICSI/IVF)	1.646 (1.549-1.750)	<0.001**	2.024 (1.834-2.234)	<0.001**	1.843 (1.734-1.959)	<0.001**
Insemination method (rescue-ICSI/IVF)	1.587 (1.411-1.786)	<0.001**	1.738 (1.447-2.087)	<0.001**	1.618 (1.434-1.826)	<0.001**

ABNCL, abnormal cleavage; DC1, direct cleavage type 1; DC2, direct cleavage type 2; RC, rapid cleavage; CC, chaotic cleavage; BMI, body mass index; DOR, diminished ovarian reserve; EMT, endometriosis; COS, controlled ovarian stimulation; GnRH, gonadotropin-releasing hormone; Gn, gonadotropin; r-FSH, recombinant follicle stimulating hormone; u-FSH, urinary follicle stimulating hormone; E2, estradiol; hCG, human chorionic gonadotropin; IVF, in vitro fertilization; ICSI, intracytoplasmic sperm injection;OR, odds ratio; CI, confidence interval. * p < 0.05, ** p < 0.01.

## Discussion

This study uses large-sample analysis to investigate the factors influencing ABNCL in early embryos. Using univariate analysis, subgroup analysis, and confirmation through a generalized estimating equation model analysis, the key findings of this study indicated that EMT, unexplained infertility, a greater number of oocytes retrieved, greater initial and total Gn doses, ICSI, and rescue ICSI were risk factors for ABNCL, while a mild stimulation and a higher E2 level on hCG trigger day were associated with a reduced risk of ABNCL. Notably, beyond these associations, a clinically meaningful finding of this study is the substantial reduction in the Day 3 usable embryo rate among embryos exhibiting ABNCL, with a SMD of 0.596, indicating a moderate-to-large effect size. This observation provides further evidence supporting an association between abnormal cleavage patterns and reduced early embryonic developmental competence. The findings of this study provide evidence-based guidance for clinical consultations and personalized infertility treatments for infertility patients and also offer research support for additional studies on the underlying mechanisms that yield these results. However, given the large sample size of this study, it is important to note that some statistically significant associations may correspond to relatively modest effect sizes. Therefore, the clinical relevance of these findings should be interpreted with caution, with greater emphasis placed on the magnitude and direction of the observed effects rather than statistical significance alone.

In the present study, the proportion of ABNCL embryos resulting from patients with EMT and unexplained infertility was greater than that of from patients with tubal factor infertility. Previous studies have shown that EMT may disrupt normal steroid metabolism and biosynthesis, the response to oxidative stress, and cell cycle regulation within the ovaries (24). Increased oxidative stress, disrupted steroidogenesis, and an altered follicular microenvironment can all have negative effects on oocyte and embryo quality (25). Additionally, iron overload in the peritoneal fluid of women with EMT has been shown to compromise embryo development by disrupting mitochondrial function and triggering apoptosis and ferroptosis (26). EMT can also affect embryo gene expression. Studies have shown that the peritoneal fluid of women with EMT may contain factors that alter the expression of genes involved in embryo development. This can lead to abnormal embryo development, including arrest after fertilization and reduced blastocyst formation (27). Unexplained infertility was another risk factor for embryos exhibiting ABNCL, which could be explained by these embryos having subtle genetic, epigenetic, or metabolic alterations that are not easily detectable by conventional methods. These alterations could potentially affect embryo development (28). Importantly, our findings suggest that abnormal cleavage is not merely a morphological observation but is associated with impaired early embryonic developmental competence, as reflected by the lower Day 3 usable embryo rate in ABNCL embryos. The observed effect size (SMD = 0.596) indicates a moderate-to-large difference, supporting its potential relevance at the cleavage stage. However, this association is limited to early embryo development and should not be extrapolated to blastocyst formation or clinical outcomes, which were not assessed in the present study. Further studies incorporating downstream developmental and clinical endpoints are needed to clarify the broader implications of these findings. From a clinical perspective, optimizing gamete quality remains an important consideration in improving overall embryo developmental potential.

The present study found that the antagonist protocol was associated with a higher proportion of ABNCL compared to the agonist protocol, while the mild stimulation protocol resulted in a lower proportion. However, the absolute differences between protocols were relatively small, and thus the clinical relevance of protocol selection specifically for reducing ABNCL risk may be limited. These findings should be interpreted cautiously and not be overgeneralized to guide protocol selection in isolation. To substantiate whether these different protocols genuinely influence ABNCL, subsequent investigations, including stratified analysis or prospective randomized controlled trials, are essential for confirmation. An intriguing finding was that advanced age was associated with a lower risk of ABNCL. This appears to contradict the well-documented negative impact of age on oocyte quality. However, this paradox can be explained by the specific developmental context of our study. Our analysis was strictly confined to the window between normal fertilization and the day3 cleavage stage. Clinical embryology observations consistently indicate that the primary detrimental effects of advanced maternal age are often stage-specific: they are more pronounced prior to normal fertilization, during the fertilization process and in post-cleavage developmental stage (e.g., reduced blastocyst formation and significantly higher rates of embryonic aneuploidy). The relatively short period from normal fertilization to early cleavage may not be the most vulnerable window for age-related damage to manifest as aberrant cytokinesis. Consequently, our finding does not negate the overall adverse effect of age but rather suggests that its expression is phase-dependent. Moreover, the magnitude of this association was relatively small, further supporting the likelihood that this finding is influenced by selection bias and residual confounding rather than reflecting a true biological protective effect. In particular, this observation is likely influenced by a significant selection bias inherent in our study design, which included only oocytes that had successfully achieved normal fertilization. In addition, advanced age patients and those with diminished ovarian reserve are more frequently prescribed mild stimulation protocols, which, as our data show, are independently associated with a lower proportion of ABNCL. Therefore, the apparent ‘protective’ effect of age in our model may be partially confounded by the differential use of stimulation protocols.

In our analysis, a greater number of oocyte retrieved and higher initial and total Gn doses increased the risk of ABNCL. However, it is important to note that these associations were of modest magnitude, and their statistical significance is likely driven in part by the large sample size. Therefore, these findings should not be interpreted as evidence that higher Gn doses or oocyte yield directly cause clinically meaningful increases in abnormal cleavage risk. In line with current clinical practice at our center, mild COS protocols are typically recommended for patients of advanced age or with diminished ovarian reserve, and our findings further support the appropriateness of this individualized approach. Taking into account the initial Gn dose, total Gn dose, and number of oocytes retrieved, the results from this study indicate that a mild COS protocol could result in a lower proportion of ABNCL. A proper Gn dose is essential for achieving optimal follicular development and maturation, which directly influence the number and quality of oocytes retrieved (29, 30). Studies have shown that delivering an individualized Gn dose, guided by factors such as serum estradiol levels and follicle count, can significantly enhance the chances of achieving a successful pregnancy (30). One study involving patients with low BMI found that a lower Gn dose resulted in a higher number of fertilized oocytes and available embryos compared to higher dose, suggesting that a tailored approach to Gn administration can enhance outcomes (31). Another study found that a higher dose of Gn was associated with a decrease in embryo quality, particularly in younger women with normal ovarian reserves (32). Various studies have indicated that the relationship between Gn dose and embryo quality is complex and may vary based on individual patient characteristics such as age and ovarian reserve. Therefore, each patient’s response to Gn can vary significantly based on factors such as age, BMI, and underlying reproductive health issues; thus, an emphasis on personalized approaches to and individualized protocols for ovarian stimulation is paramount. Taken together, these results reinforce the importance of individualized stimulation strategies, while also highlighting that Gn-related parameters alone are unlikely to be strong independent predictors of ABNCL. Furthermore, the study demonstrated that elevated E2 levels on the hCG trigger day were associated with a reduced risk of ABNCL. Although statistically significant, the protective association between higher E2 levels and ABNCL was modest in magnitude and likely reflects overall follicular competence rather than a direct causal effect of estradiol. This finding may be attributed to the central role of estradiol in regulating the follicular microenvironment and oocyte developmental competence (33). Higher E2 concentrations generally reflect coordinated follicular growth and active granulosa cell steroidogenesis (34), both of which are critical for the synchronized nuclear and cytoplasmic maturation of oocytes. At the molecular level, estradiol modulates mitochondrial function, oxidative stress, and cell cycle–related pathways through estrogen receptor–mediated signaling (35), thereby supporting oocyte maturation and developmental competence. In addition, E2 promotes granulosa cell survival and upregulates luteinizing hormone receptor expression (36), thereby enhancing follicular responsiveness to the hCG trigger and facilitating timely meiotic resumption (37). Collectively, these mechanisms may contribute to improved oocyte quality and a reduced likelihood of abnormal cleavage. However, this apparent protective effect likely reflects enhanced oocyte competence rather than a direct effect of supraphysiological E2 levels on the embryo. Importantly, these interpretations are confined to early embryonic development and should not be extended to infer downstream effects on blastocyst quality, implantation, or live birth outcomes.

Regarding *in vitro* fertilization methods, the proportion of ABNCL embryos resulting from patients who underwent ICSI or rescue ICSI was higher than that from patients who underwent conventional IVF. Male factor infertility did not appear to be a risk factor for ABNCL in this study, indicating that ICSI may impact embryos’ developmental capacity. ICSI is known to be associated with improved outcomes compared to IVF in patients with severe male factor infertility. In our center, ICSI is employed for cases of male factor infertility and for patients who have experienced previous failure of or low fertilization with conventional IVF. Furthermore, the need for rescue ICSI was determined based on the extrusion of the second polar body on the day of conventional IVF, and it was immediately performed within a specific time window to avoid total fertilization failure. A large cohort study suggested that ICSI has no benefit regarding pregnancy outcome in all autologous ovarian response cycles with normal sperm parameters, despite improved fertilization rates (38). A study that examined the effects of IVF and ICSI on high-quality blastocyst development in a sibling oocyte cohort found that the IVF group achieved a higher blastulation rate per 2PN compared to the ICSI group. However, this difference was not evident when considering the number of oocytes designated for each procedure (39). The mechanism by which ICSI affects embryo quality is related to the direct injection of a selected sperm into the oocyte. This can bypass certain barriers to fertilization and potentially improve the chances of successful embryo development. A previous study showed that, while ICSI can result in the selection of a good-quality motile spermatozoon, it does not always guarantee better pregnancy and live birth rates compared to conventional IVF (40). Studies have shown that rescue ICSI can be a safe and effective means of decreasing total IVF fertilization failure. Although the multi-PN fertilization rate may be higher, this approach can still result in comparable clinical pregnancy rates and similar rates of congenital birth defects compared to other methods (41). Importantly, embryos derived from rescue ICSI represent a biologically distinct subgroup, as they originate from oocytes that failed to achieve fertilization following conventional IVF. This underlying fertilization failure may reflect intrinsic oocyte or sperm dysfunction, which could independently influence early embryonic cleavage patterns and contribute to the observed increase in ABNCL risk. Therefore, the association between rescue ICSI and ABNCL should be interpreted with caution, as it may not solely reflect a procedural effect. Similarly, embryos derived from delayed IVF insemination constitute another biologically distinct subgroup. The timing of insemination may affect oocyte competence and subsequent embryonic development, potentially confounding the relationship between insemination method and abnormal cleavage. Unlike previous studies, our study found that ICSI and rescue ICSI significantly increased the rate of ABNCL. The observed increased risk of ABNCL associated with ICSI necessitates distinguishing between a procedural effect and confounding by the underlying male factor indication. To explore this, we conducted a stratified analysis based on semen quality in a related cohort (data from a separate study). While ICSI consistently conferred a higher ABNCL risk compared to conventional IVF across all strata, including normozoospermia, the strength of this association was not static, and it attenuated with progressively poorer semen parameters. This pattern indicates that semen quality acts as an effect modifier rather than a simple confounder. One plausible interpretation is that in severe male factor infertility, the inherent sperm pathology itself is the primary driver of early developmental abnormalities, potentially masking any incremental risk attributable to the ICSI procedure. In contrast, in the context of normal semen parameters, the persistent risk elevation may render a procedural contribution—such as the bypass of physiological sperm-oocyte interactions or mechanical stress—more discernible. Therefore, future prospective studies focusing on couples without male factor infertility are warranted to validate these findings. Overall, these findings suggest that the choice of insemination method should continue to be guided primarily by clinical indications rather than concerns regarding modest differences in ABNCL risk.

### Limitations

Our study is the largest study to analyze factors influencing ABNCL embryos to date. We utilized 3 years of continuous data from a single center, and the assisted reproductive protocols, clinical factors, laboratory conditions, and personnel were consistent throughout this time period, which ensures the reliability of the results. However, the retrospective nature of our study presents some limitations, even after we excluded confounding factors like PGT cycles, frozen-thawed oocyte cycles, and cycles involving donor sperm. Of note, we excluded all oocytes and embryos from which annotation of cleavage patterns was not available, focusing on those derived from normal fertilized embryos and with annotation of cleavage patterns. This approach may have obscured some factors associated with cycles that exhibited lower mature oocyte rates and normal fertilization rates. Therefore, our findings apply to normally fertilized oocytes from this specific population, rather than all patients or cycles in the initial population. Additionally, our retrospective analysis did not consider all parameters of the 138,178 embryos that were assessed. The data were retrieved from original records, and missing data were not analyzed. Furthermore, parameters such as paternal age and sperm DNA fragmentation, which are known to affect embryo quality, were not analyzed and warrant investigation in future studies focusing on unexplained infertility. In addition, although a comparative analysis of the developmental potential among different abnormal cleavage subtypes would be clinically meaningful, such an analysis was not feasible in the present study. The most appropriate indicator for evaluating developmental potential in this context would be the blastocyst formation rate; however, not all included patients underwent extended embryo culture to the blastocyst stage. Moreover, in routine clinical practice, some patients choose to culture only a portion of their embryos to the blastocyst stage rather than all available embryos, which further complicates the interpretation of blastocyst formation outcomes and may introduce additional selection bias. Therefore, the current dataset was not ideally suited to reliably compare developmental potential across different abnormal cleavage subtypes. More importantly, the absence of blastocyst-stage and clinical outcome data limits our ability to determine whether ABNCL has a sustained impact beyond the cleavage stage. As such, any potential influence of ABNCL on implantation, pregnancy, or live birth outcomes remains speculative and should be interpreted with caution. Future studies based on PGT populations, in which blastocyst culture is performed more routinely and consistently, may provide more convincing evidence on this issue. Nevertheless, our comprehensive analysis of factors affecting embryo ABNCL based on a large sample size and robust statistical analysis provides strong evidence-based support for clinical consultation and individualized treatment. This study also lays the groundwork for future analyses of individual factors influencing ABNCL and their potential mechanisms.

## Conclusions

In summary, this large TLI-based cohort demonstrates that ABNCL is common in early human embryos and is shaped by both patient characteristics and treatment-related variables. Endometriosis, unexplained infertility, increased oocyte yield, and the use of ICSI or rescue ICSI were independently associated with higher ABNCL risk, whereas mild stimulation and higher E2 levels on the trigger day were protective. These findings highlight the need for individualized COS and insemination strategies to support optimal early embryonic cleavage and improve ART decision-making, although their implications are limited to cleavage-stage outcomes and should not be extrapolated to later developmental or clinical endpoints.

## Data Availability

The raw data supporting the conclusions of this article will be made available by the authors, without undue reservation.
